# Tuina therapy alleviates neuropathic pain by modulating the CX3CL1/CX3CR1 axis to inhibit degradation of the perineuronal nets

**DOI:** 10.3389/fnmol.2026.1860375

**Published:** 2026-07-02

**Authors:** Yukui Tian, Cheng Wang, Qingguang Zhu, Lei Guo, Xue Bai, Gulaisar Aikebaier, Xiaofeng Cui, Zhiwei Wu, Junchang Liu

**Affiliations:** 1 School of Traditional Chinese Medicine, Xinjiang Medical University, Ürümqi, Xinjiang, China; 2 Traditional Chinese Medicine Hospital of Xinjiang Uygur Autonomous Region, Ürümqi, Xinjiang, China; 3 Institute of Traditional Chinese Medicine of Xinjiang Uygur Autonomous Region, Ürümqi, Xinjiang, China; 4 Institute of Tuina, Shanghai Academy of Traditional Chinese Medicine, Shanghai, China; 5 Yueyang Hospital of Integrated Traditional Chinese and Western Medicine, Shanghai University of Traditional Chinese Medicine, Shanghai, China; 6 Guanghua Hospital Affiliated to Shanghai University of Traditional Chinese Medicine, Shanghai, China

**Keywords:** CX3CL1/CX3CR1, neuropathic pain, perineuronal nets, phagocytosis, Tuina

## Abstract

**Introduction:**

Tuina, a traditional Chinese non-pharmacological therapy, is effective in treating neuropathic pain (NP). However, its underlying molecular mechanisms remain elusive. The degradation of perineuronal nets (PNNs) via the CX3CL1/CX3CR1 axis is a key driver of central sensitization. This study investigates whether Tuina exerts analgesia by modulating this specific neuro-immune axis to preserve PNNs integrity in a rat model of chronic compression of the dorsal root ganglion (CCD).

**Methods:**

Sprague–Dawley rats were subjected to CCD surgery to establish the NP model. To validate the role of PNNs, a subset of rats received intrathecal chondroitinase ABC (chABC). To explore Tuina’s mechanism, rats were divided into Sham, Model, Tuina, CX3CR1 inhibitor (AZD8797), and Tuina + AZD8797 groups. Standardized Tuina intervention or inhibitor administration was performed from days 4 to 14 post-surgery. Pain behaviors (PWT and PWL) were evaluated. Spinal dorsal horn tissues were analyzed using transmission electron microscopy, Western blotting, immunofluorescence, and flow cytometry.

**Results:**

CCD induced severe mechanical allodynia and thermal hyperalgesia, accompanied by the structural disruption of spinal PNNs—indicated by a significant loss of WFA-binding glycosaminoglycans despite unchanged total aggrecan core protein levels—and ultrastructural neuronal damage. Mechanistically, CCD upregulated the CX3CL1/CX3CR1/PI3K/AKT cascade, which was associated with increased markers of microglial phagocytosis (upregulated TREM2 and LAMP1), as well as Ca^2+^ influx and c-FOS accumulation. Tuina intervention attenuated these pathological alterations, significantly restoring WFA expression and suggesting a preservation of PNNs structural integrity. Furthermore, the combined treatment (Tuina + AZD8797) yielded an enhanced analgesic and biochemical recovery compared to either monotherapy.

**Conclusion:**

Tuina produces robust central analgesia by suppressing the CX3CL1/CX3CR1-PI3K/AKT signaling pathway. This suppression restores microglial phagocytic homeostasis and crucially protects the glycosaminoglycan lattice of PNNs against excessive degradation, thereby alleviating neuropathic central sensitization.

## 1 Introduction

Neuropathic pain (NP) is among the most debilitating pain conditions, arising from various etiologies such as diabetic painful neuropathy, traumatic neuropathy, herpes zoster, intervertebral disc herniation, multiple sclerosis, spinal cord injury, traumatic brain injury, and stroke (Attal et al., 2023). The primary clinical manifestations of NP include spontaneous pain and hyperalgesia. Patients often describe these sensory deficits as electric shock-like, stabbing, burning, numbing, or pulsating sensations. Additionally, NP frequently leads to significant affective comorbidities, including sleep disturbances, depression, and anxiety. Epidemiological studies estimate that NP affects approximately 7 to 10% of the adult population and is increasingly recognized as a prevalent underlying cause of chronic pain (van Hecke et al., 2014).

Central sensitization (CS) represents a fundamental mechanism underlying aberrant nociceptive processing in patients with chronic pain. Defined as a state of neuronal hyperexcitability within spinal and supraspinal pathways, CS plays a critical role in both the initiation and maintenance of neuropathic pain (NP) (Ma et al., 2024). Emerging evidence indicates that the spinal dorsal horn (SDH) serves as a pivotal hub for the transmission and modulation of nociceptive information (Li et al., 2022). Specifically, Lamina I of the SDH is responsible for receiving and integrating peripheral nociceptive signals before transmitting them to supraspinal centers, playing a central role in pain regulation (Tsuda et al., 2017). Recent investigations have identified a distinct population of spinoparabrachial projection neurons within the SDH that are ensheathed by perineuronal nets (PNNs) and are integral to the encoding of pain information (Tansley et al., 2022). Microglia, the resident immune sentinels of the central nervous system (CNS), play a pivotal role in neuroplasticity. Following peripheral nerve injury, activated microglia mediate the phagocytic erosion of perineuronal nets (PNNs) (Cheung et al., 2024). This structural degradation compromises the inhibitory brake on projection neurons, thereby precipitating neuronal hyperexcitability and amplified nociceptive transmission. This pathological cascade is orchestrated by the CX3CL1/CX3CR1 signaling axis (Bago-Mas et al., 2025). Activation of this axis drives neuroinflammatory responses, promotes pro-inflammatory cytokine release and neuronal Ca^2+^ influx, ultimately perpetuating a state of pain hypersensitivity (Arreola et al., 2021; Liu et al., 2023).

As a quintessential modality of traditional Chinese non-pharmacological medicine, Tuina is extensively practiced in clinical settings and has demonstrated significant therapeutic efficacy in managing diverse pain conditions, particularly neuropathic pain (NP) (Liu et al., 2022; Hongye et al., 2023). Previous investigations have substantiated that Tuina intervention significantly attenuates mechanical allodynia and thermal hyperalgesia in NP rat models (Song et al., 2018). Mechanistically, it modulates the phenotypic shift of microglia from the pro-inflammatory M1 state to the anti-inflammatory M2 state, thereby downregulating the expression of pro-inflammatory cytokines—specifically Interleukin-6 (IL-6), Interleukin-1β (IL-1β), and Tumor Necrosis Factor-alpha (TNF-*α*)—and effectively reversing the neuroinflammatory cascade (Zhiwei et al., 2024).

However, direct evidence remains elusive regarding whether Tuina exerts its central analgesic effects by modulating the CX3CL1/CX3CR1 axis to influence microglial phagocytic activity, thereby interfering with the degradation of perineuronal nets (PNNs). Consequently, in the present study, we established a rat model of neuropathic pain and administered an 11-day Tuina intervention. Our objective was to elucidate whether the analgesic efficacy of Tuina is mediated through the regulation of the CX3CL1/CX3CR1–microglia–PNNs signaling axis, which in turn modulates spinal central sensitization and alleviates hyperalgesia.

## 2 Methods and materials

### 2.1 Ethical approval and animal care

This study was approved by the Institutional Animal Care and Use Committee (IACUC) of Xinjiang Medical University on February 13, 2025 (Approval No. IACUC-20250213-01). All procedures were conducted according to the Guide for the Care and Use of Laboratory Animals as outlined by the US National Institutes of Health and adhered to the Animal Research Reporting standards: *In vivo* Experiments (ARRIVE) guidelines (Percie du Sert et al., 2020; Ashall et al., 2023).

### 2.2 Animals

Healthy male Sprague–Dawley (SD) rats (SPF grade), aged 8 weeks and weighing 200 ± 20 g, were procured from the Animal Experiment Center of Xinjiang Medical University. Animals were maintained in a temperature- and humidity-controlled facility (24 ± 3 °C; 40–60% humidity) on a 12-h light/dark cycle with *ad libitum* access to standard rodent chow and water. Acclimatization was performed for a minimum of 7 days before experimentation.

### 2.3 Experimental design

To elucidate the mechanisms underlying central sensitization and evaluate the therapeutic targets of Tuina, the *in vivo* studies were structured into two parts. Experiment 1: To verify the role of perineuronal net (PNNs) degradation in hyperalgesia, rats were randomized into three groups (*n* = 12/group): Sham, chronic compression of the dorsal root ganglion (Model), and chondroitinase ABC (chABC). Experiment 2: To investigate the analgesic mechanism of Tuina via the CX3CR1 axis, a separate cohort was randomly allocated to five groups (*n* = 12/group) using a computer-generated random number sequence: Sham, Model, Tuina, CX3CR1 inhibitor (AZD8797), and Tuina + AZD8797 (TA).

### 2.4 Establishment of the CCD model

The CCD model was established as previously described. Briefly, rats were fasted and water-deprived for 8 h prior to surgery. Anesthesia was induced via intraperitoneal injection of 2% pentobarbital sodium (30 mg/kg; Sigma-Aldrich, St. Louis, MO, USA). The surgical site was shaved and disinfected with povidone-iodine, and rats were secured in a prone position. A unilateral incision was made at the L4–L6 level, and the paraspinal muscles were dissected to expose the L4–L5 intervertebral foramen. Two L-shaped stainless steel rods (4 mm × 0.6 mm) were inserted into the foramen at a 30° angle to the spine to compress the L4 and L5 DRGs (Yao et al., 2022). Post-operative X-ray imaging was performed using an InAlyzer system (MEDIKORS, Korea) to verify the accurate positioning of the stainless steel rods (parameters: current 1.25 mA/1.0 mA; voltage 55 kV/80 kV).

### 2.5 Tuina intervention and drug administration

#### 2.5.1 chABC administration (Experiment 1)

To specifically degrade PNNs in Experiment 1, rats in the chABC group received intrathecal injections of chABC (6 μL, 1 U/mL) every other day for a total of six doses, commencing on post-operative day 4 (D4) and concluding on post-operative day 14 (D14). The chABC group did not receive any Tuina or other interventions.

#### 2.5.2 Tuina intervention and drug administration (Experiment 2)

Commencing on D4 post-surgery, rats in the Tuina and TA groups were restrained in a prone position with the ipsilateral hind limb fully exposed. Tuina manipulation, consisting of thumb pressing and kneading, was performed on the ipsilateral acupoints Yinmen (BL37), Weizhong (BL40), and Chengshan (BL57). This intervention was administered once daily for 5 min per acupoint, with an interval of less than 1 min between acupoints, over a period of 11 consecutive days. To ensure consistency and eliminate inter-operator variability, all Tuina manipulations were performed by a single, well-trained operator. To standardize the procedure, a tactile pressure sensor (FingerTPS, Pressure Profile System, CA, USA) was utilized to maintain a constant stimulation pressure of 5 ± 0.5 N at a frequency of 2 Hz. Prior to each session, rats were habituated to the fixation apparatus for 15 min to minimize environmental stress (Wu et al., 2023). To eliminate bias arising from handling, animals in the Sham and Model groups underwent identical restraint and habituation protocols for the same duration but received no therapeutic manipulation.

In the AZD8797 and TA groups, intraperitoneal (i.p.) administration of the CX3CR1 inhibitor AZD8797 (80 μg/kg) was initiated on D4 post-modeling, with a frequency of once daily (Chen et al., 2020). For animals receiving combined therapy (TA group), the interval between Tuina manipulation and AZD8797 injection was strictly maintained at greater than 2 h to prevent immediate interaction effects.

### 2.6 Behavioral tests

Paw Withdrawal Threshold (PWT) and Paw Withdrawal Latency (PWL) were assessed at baseline (pre-operation) and on post-operative days 4, 7, 10, and 14. For PWT assessment, rats were acclimatized in individual Plexiglas cages (20 × 10 × 20 cm) on an elevated mesh floor. An electronic Von Frey aesthesiometer (Model 38,450, UGO Basile, Italy) was used to apply mechanical stimulation to the plantar surface of the ipsilateral hind paw, and the force intensity inducing paw withdrawal was recorded five times with a 1-min inter-stimulus interval (Zhiwei et al., 2024). Subsequently, PWL was evaluated using a hot plate analgesia meter (SY-YLS-6BS, Shanghai Shaoyi Biotechnology, China) maintained at 50 °C. The latency to the first sign of nociception (paw retraction, shaking, or licking) was recorded (Hargreaves et al., 1988). A cut-off time of 20 s was imposed to prevent tissue damage, with a 5-min interval between trials. At each time point, measurements were performed in quintuplicate; after excluding the maximum and minimum values, the mean of the remaining three readings was calculated as the final result (Wu et al., 2023).

### 2.7 Transmission electron microscopy (TEM)

TEM was performed to examine the ultrastructure of neurons in the spinal dorsal horn. Tissue samples were prefixed with 2.5% glutaraldehyde and postfixed with 1% osmium tetroxide, followed by dehydration through a graded acetone series. Infiltration was conducted using mixtures of acetone and Epon-812 embedding medium at ratios of 3:1, 1:1, and 1:3, respectively, prior to embedding in pure Epon-812. After localizing the target region via semithin sectioning and optical microscopy, ultrathin sections (60–90 nm) were cut, mounted on copper grids, and double-stained with uranyl acetate (10–15 min) and lead citrate (1–2 min). Images were acquired using a JEM-1400FLASH transmission electron microscope (JEOL, Tokyo, Japan).

### 2.8 Immunofluorescence

Spinal cord sections were deparaffinized, rehydrated, and subjected to heat-induced antigen retrieval utilizing a citrate buffer (pH 6.0). Endogenous peroxidases were quenched with 3% H_2_O_2_, and sections were blocked with 3% BSA (Boster Biological Technology, Wuhan, China; AR1006). For immunofluorescence labeling, sections were incubated overnight at 4 °C with the following primary antibodies: anti-c-FOS (Affinity Biosciences, Jiangsu, China; AF5354, 1:500), anti-WFA (Vector Laboratories, Newark, CA, USA; FL-1351-2, 1:1000), or anti-aggrecan (Affinity; DF7561, 1:2000) (Grycz et al., 2022). Subsequently, sections were incubated with HRP-conjugated goat anti-rabbit (Servicebio, Wuhan, China; GB23303) or anti-mouse (Servicebio; GB21301) secondary antibodies for 50 min at room temperature. Signal amplification was achieved using Tyramide Signal Amplification (TSA) fluorophores (1:500) for 15 min: iF488-Tyramide (Servicebio; G1231) for c-FOS and WFA; and iF594-Tyramide (Servicebio; G1242) for aggrecan. For co-labeling, a sequential TSA protocol was employed. Antibody complexes from the preceding cycle were completely eluted by heating the sections at 95 °C in citrate buffer for 30 min before repeating the blocking and staining steps for the next target to prevent cross-reactivity. Nuclei were counterstained with DAPI (Fusheng, Shanghai, China; FSM0180-100 T). Slides were mounted using an anti-fade mounting medium (Biosharp, Beijing, China; BL701A) and imaged via an Olympus BX53 fluorescence microscope (Song et al., 2018).

### 2.9 Western blotting

Ipsilateral spinal dorsal horn tissues were homogenized in liquid nitrogen, lysed in RIPA buffer supplemented with protease and phosphatase inhibitors, and centrifuged to obtain the supernatant. The protein concentration was quantified using a BCA assay. Samples were denatured with 5 × SDS-PAGE loading buffer and subjected to SDS-PAGE (stacking gel: 80 V; separating gel: 100 V; duration: 90 min). Proteins were transferred to PVDF membranes (0.22 μm for TREM2; 0.45 μm for other proteins) using optimized transfer times: 60 min for TREM2, Lamp1, AKT, p-AKT, CX3CR1, and Actin, and 90 min for PI3K, p-PI3K, and CX3CL1. After blocking with 5% skim milk for 1 h, membranes were incubated overnight at 4 °C with specific primary antibodies, followed by incubation with HRP-conjugated secondary antibodies for 1 h at room temperature. Protein bands were visualized using ECL substrate on a ChemiScope mini system (Song et al., 2018; Wang Z. et al., 2024).

### 2.10 Flow cytometry

To evaluate intracellular Ca^2+^, spinal dorsal horn tissues were enzymatically digested with 0.2 mg/mL collagenase IV and 0.05 mg/mL DNase I at 37 °C (220 rpm) for 1 h. Following filtration (70-μm) and centrifugation (400 × g, 5 min), red blood cells were lysed. The cells were then washed and resuspended in PBS (Solarbio, Beijing, China; P1010) at 1 × 10^6^ cells/mL, and incubated with 5 μm Fluo-4 a.m. (Beyotime, Shanghai, China; S1061S) for 20 min at 37 °C in the dark. For complete de-esterification, cells were cultured in PBS containing 1% fetal bovine serum (Servicebio, Wuhan, China; G1209) for 40 min. After three washes, cells were resuspended in HEPES buffer (1 × 10^5^ cells/mL), equilibrated for 10 min at 37 °C, and analyzed using the FITC channel of a flow cytometer.

### 2.11 Statistical analysis

To minimize bias, behavioral analyses were performed by investigators blinded to group allocation. Although the operator performing the Tuina intervention could not be blinded to the treatment assignments, a strict blinding protocol was maintained for all subsequent assessments. Specifically, tissue processing, Western blot analysis, immunofluorescence imaging and quantifications, as well as flow cytometry data analysis, were all independently conducted by investigators who were strictly blinded to the experimental group assignments. Statistical analyses were performed using SPSS 21.0, and graphs were generated with GraphPad Prism 9.0. For all quantitative analyses, all reported *n* values refer to independent animals rather than technical replicates. Data normality was assessed using the Shapiro–Wilk test, and homogeneity of variance was evaluated using Levene’s test. Normally distributed data were presented as mean ± SD and analyzed using one-way ANOVA. For post-hoc comparisons, the Least Significant Difference (LSD) test was applied when variances were homogeneous, whereas Dunnett’s T3 test was utilized when the assumption of equal variances was violated. Non-normally distributed data were expressed as median (IQR) and analyzed using the Kruskal-Wallis H test. Behavioral time-course data were analyzed using repeated-measures ANOVA. Mauchly’s test was used to assess the assumption of sphericity; if this assumption was violated, the Greenhouse–Geisser correction was applied. This was followed by LSD post-hoc tests for between-group comparisons at each specific time point. *p* < 0.05 was considered statistically significant.

## 3 Results

### 3.1 Degradation of PNNs in the spinal dorsal horn is sufficient to induce mechanical and thermal hyperalgesia

**Figure 1:**
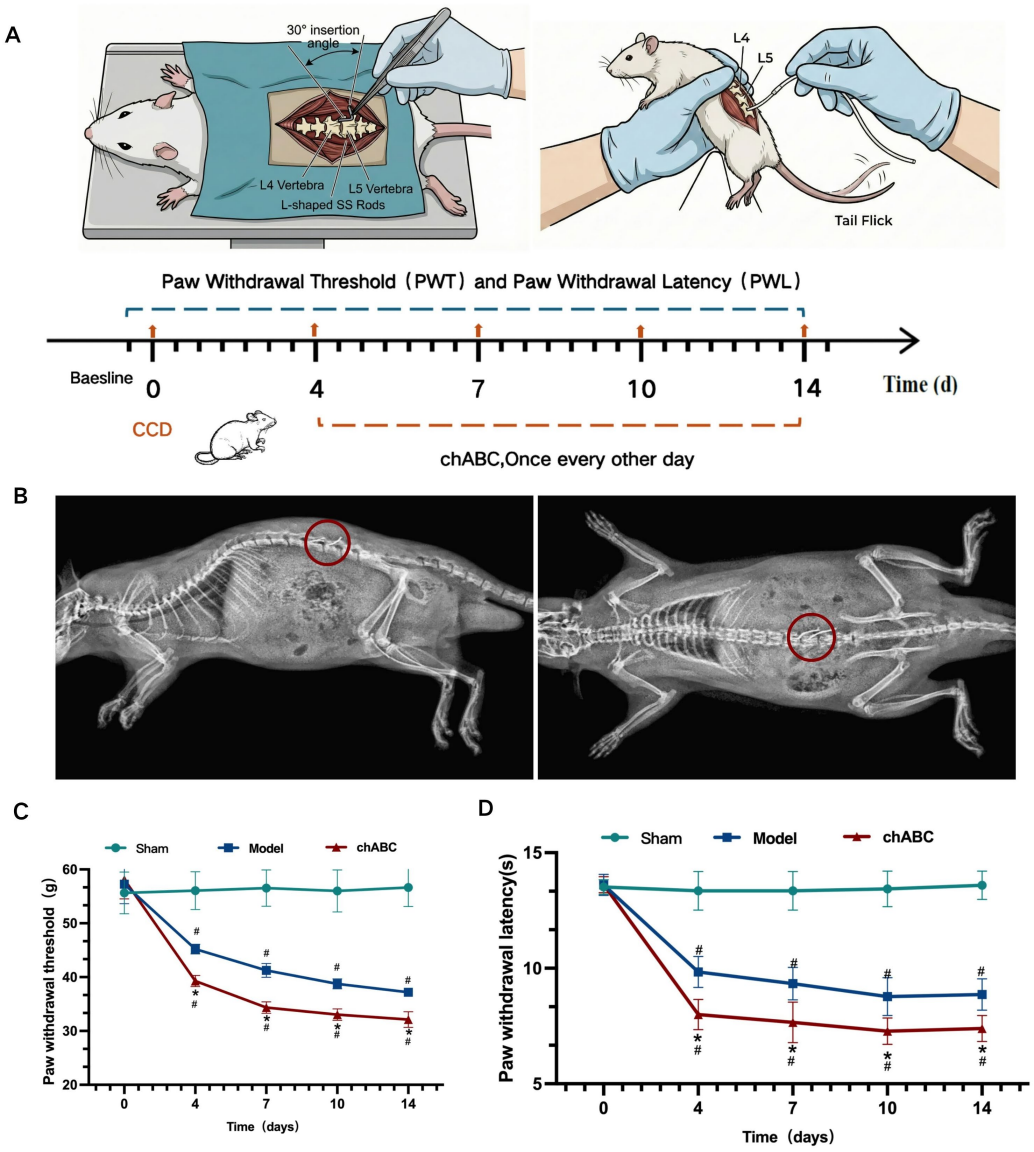
Degradation of PNNs in the spinal dorsal horn is sufficient to induce mechanical and thermal hyperalgesia. **(A)** Schematic timeline of the experimental design for Experiment 1 (chABC intervention). **(B)** Representative X-ray images confirming the correct placement of the L-shaped stainless steel rods compressing the L4 and L5 dorsal root ganglia. **(C,D)** Behavioral assessments of the paw withdrawal threshold [PWT **(C)**] and paw withdrawal latency [PWL **(D)**] at baseline and on post-operative days 4, 7, 10, and 14. Data are presented as mean ± SD (*n* = 12) (compared with sham group, *^#^p* < 0.001; compared with CCD model group, *p < 0.001). CCD, chronic compression of dorsal root ganglion model. Data were analyzed using repeated-measures ANOVA followed by LSD post-hoc tests for between-group comparisons at each time point.

To elucidate the role of perineuronal nets (PNNs) in nociceptive sensitization, we established a rat model of chronic compression of the dorsal root ganglion (CCD) and included a parallel mechanistic cohort receiving isolated intrathecal injections of chondroitinase ABC (chABC) (Figure 1A). Radiographic imaging confirmed the precise insertion of L-shaped stainless steel rods into the L4 and L5 intervertebral foramina, achieving effective compression of the dorsal root ganglia (Figure 1B). Behavioral assessments revealed that rats in the Sham group maintained relatively stable paw withdrawal latency (PWL) and paw withdrawal threshold (PWT) baselines throughout the postoperative observation period. In contrast, compared to the Sham group, CCD rats exhibited significantly reduced PWT and PWL on days 4, 7, 10, and 14 post-surgery (*p* < 0.001). Notably, rats receiving isolated intrathecal chABC injections displayed a similarly profound decline in both PWT and PWL, closely mirroring the hyperalgesic trajectory of the CCD model group (Figures 1C,D). These findings suggest that PNNs degradation is a critical driver in the pathogenesis of pain sensitization.

### 3.2 chABC specifically cleaves the glycosaminoglycan chains of spinal PNNs

**Figure 2:**
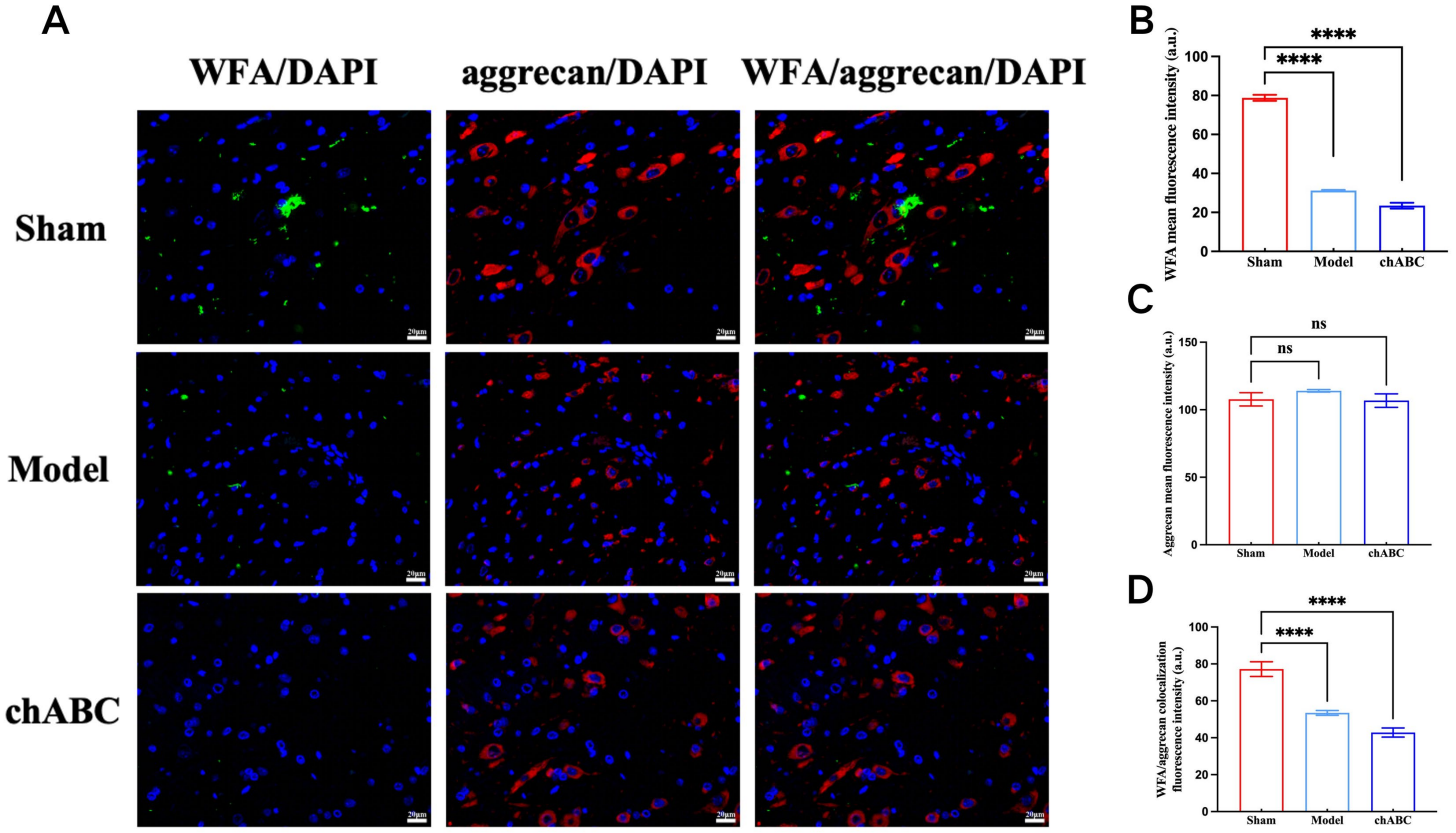
chABC specifically cleaves the glycosaminoglycan chains of PNNs without altering core proteins. **(A)** Representative immunofluorescence images of WFA (green, indicating glycosaminoglycan chains), aggrecan (red, indicating core proteins), and DAPI (blue, nuclei) in the spinal dorsal horn. Scale bar = 20 μm. **(B–D)** Quantitative analysis of the mean fluorescence intensity for WFA **(B)**, aggrecan **(C)**, and their co-localization **(D)**. Data are presented as mean ± SD (*n* = 8). Compared with the sham group, ****p < 0.0001, ^ns^*p* > 0.05. Statistical analysis was performed using one-way ANOVA followed by Tukey’s post-hoc test.

To validate the specific *in vivo* enzymatic cleavage by chABC, immunofluorescence was employed to co-localize the glycosaminoglycan (GAG) side chains (labeled with WFA) and the core protein (labeled with aggrecan) of perineuronal nets (PNNs) (Figure 2A). Quantitative analysis revealed a significant reduction in the mean fluorescence intensity of WFA in the spinal dorsal horns of both the chABC and Model groups relative to the Sham group (*p* < 0.05, Figure 2B), indicating a severe disruption of the PNNs lattice. Conversely, the fluorescence intensity of aggrecan remained unaltered across all experimental groups (*p* > 0.05, Figure 2C), a finding further corroborated by co-localization mapping (Figure 2D). These results indicate that both the pathological CCD state and chABC intervention primarily compromise the barrier function of PNNs by selectively cleaving their glycan chains, rather than directly degrading the aggrecan core protein.

### 3.3 PNNs degradation triggers intracellular calcium dyshomeostasis and neuronal ultrastructural damage

**Figure 3:**
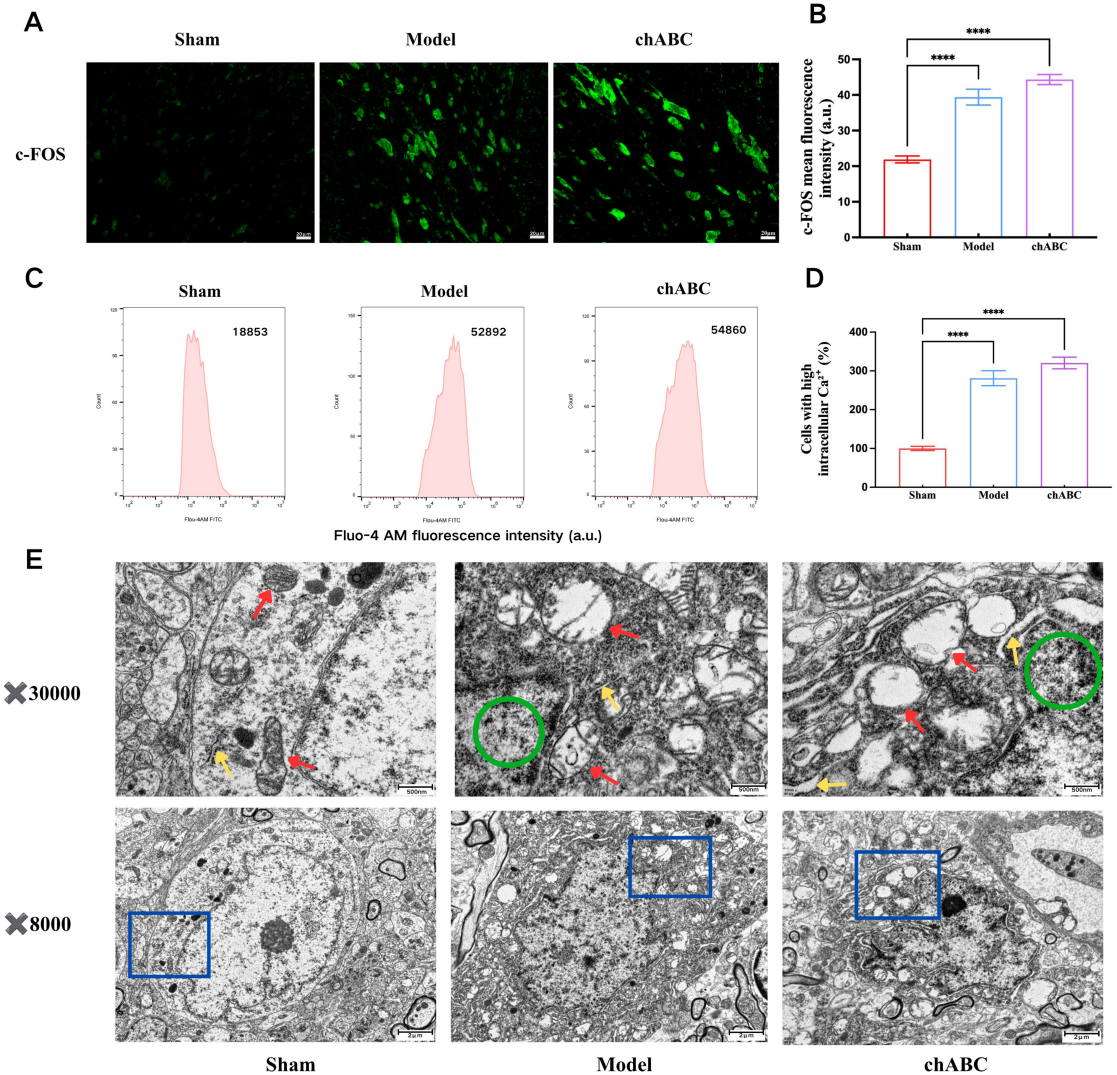
PNNs degradation triggers intracellular calcium dyshomeostasis and neuronal ultrastructural damage. **(A,B)** Representative immunofluorescence images and quantitative analysis of the neuronal activation marker c-FOS (green) in the spinal dorsal horn. Scale bar = 20 μm. **(C,D)** Representative flow cytometry plots and quantitative analysis showing the percentage of spinal cells with high intracellular Ca^2+^ levels (Fluo-4 a.m.). **(E)** Representative transmission electron microscopy (TEM) images of neuronal ultrastructure in the spinal dorsal horn. Red arrows indicate mitochondria; yellow arrows indicate rough endoplasmic reticulum; green circles indicate nuclear chromatin. Scale bars: 500 nm in the upper row; 2 μm in the lower row. Data are presented as mean ± SD (*n* = 8). ****p < 0.0001 vs. Sham group. Statistical analysis was performed using one-way ANOVA followed by Tukey’s post-hoc test.

Disruption of the PNNs matrix typically compromises its neuroprotective capacity. Immunofluorescence analysis revealed basal levels of c-FOS expression in the spinal cord of the Sham group; conversely, both the Model and chABC groups exhibited a dramatic upregulation in c-FOS expression, as evidenced by significantly enhanced mean fluorescence intensity (*p* < 0.0001, Figures 3A,B). Flow cytometry further suggest that the proportion of spinal dorsal horn cells with high intracellular Ca^2+^ was significantly elevated in the Model and chABC groups relative to the Sham group (*p* < 0.05, Figures 3A,B), indicating that PNNs degradation precipitates neuronal calcium overload (Figures 3C,D). Furthermore, transmission electron microscopy (TEM) revealed intact neuronal ultrastructures in the Sham group, characterized by continuous nuclear envelopes, evenly distributed chromatin, normal flattened cisternae of the rough endoplasmic reticulum (rER), and well-preserved mitochondria with distinct cristae and homogeneous matrices. In striking contrast, neurons in both the CCD Model and chABC groups displayed marked pathological alterations. These included abnormal nuclear pyknosis with highly condensed chromatin, as well as severe mitochondrial swelling and vacuolization characterized by matrix loss, effacement of cristae, and occasional outer membrane rupture. Concurrently, mild dilation of the rER was observed (Figure 3E). Collectively, these findings indicate that the degradation of PNNs directly drives aberrant neuronal hyperexcitability and ultrastructural damage.

### 3.4 Tuina alleviates CCD-induced hyperalgesia and ameliorates neuronal ultrastructural damage

**Figure 4:**
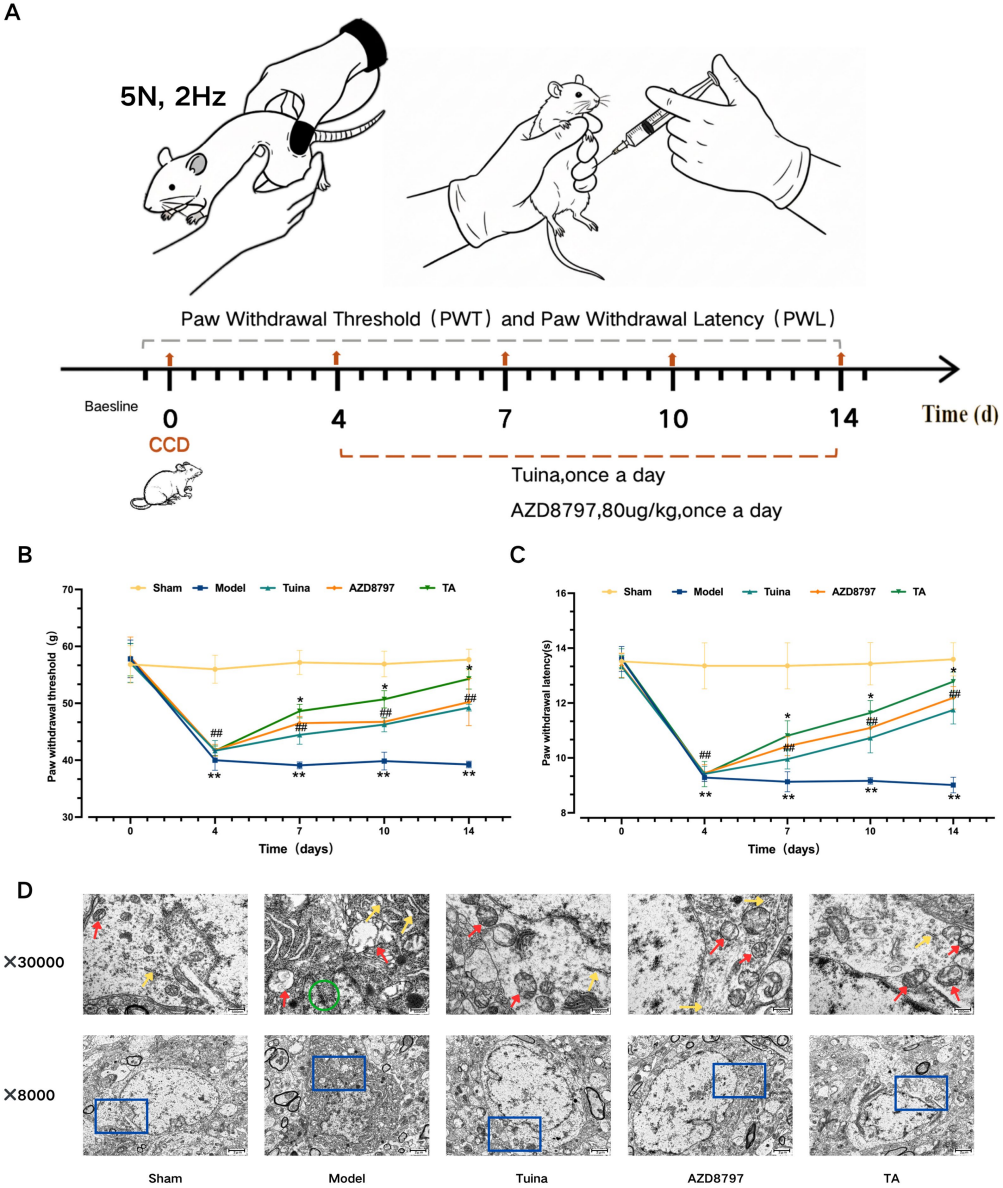
Tuina alleviates CCD-induced hyperalgesia and ameliorates neuronal ultrastructural damage. **(A)** Schematic diagram of the experimental timeline. **(B,C)** Time course of Paw Withdrawal Threshold (PWT) and Paw Withdrawal Latency (PWL) across groups. Data are expressed as mean ± SD (*n* = 8). **(D)** Representative TEM images showing the protective effects of Tuina and AZD8797 on neuronal ultrastructure (nuclei, mitochondria, and endoplasmic reticulum) in the spinal dorsal horn. The red arrow indicates mitochondria; the yellow arrow indicates the rough endoplasmic reticulum; the green circle indicates nuclear chromatin condensation. Scale bars: 500 nm in the upper row; 2 μm in the lower row. Data were analyzed using repeated measures ANOVA followed by LSD post-hoc tests. *^##^p* < 0.01 vs. Sham group; **p < 0.01 vs. Model group; *p < 0.01 vs. Tuina group. Data were analyzed using repeated-measures ANOVA followed by LSD post-hoc tests for between-group comparisons at each time point.

Having established the role of PNNs degradation in central sensitization, we evaluated the therapeutic efficacy of Tuina and its functional association with the CX3CR1 receptor (Figure 4A). Behavioral assessments revealed that rats in the Sham group maintained relatively stable paw withdrawal latency (PWL) and paw withdrawal threshold (PWT) baselines throughout the postoperative observation period. In contrast, CCD rats exhibited a precipitous decline in both PWL and PWT compared to the Sham group (*p* < 0.01), which reached a nadir on day 7 (*p* < 0.01) and persisted until day 14 (*p* < 0.01), confirming the successful induction of robust nociceptive hypersensitivity. Strikingly, Tuina intervention significantly reversed these behavioral deficits, yielding markedly higher PWT and PWL values than those of the CCD group on days 7, 10, and 14 post-surgery (*p* < 0.01). While the isolated administration of the CX3CR1 inhibitor (AZD8797) produced an analgesic profile comparable to that of Tuina, with no significant differences observed between the two groups at matched time points (*p* > 0.05), the combined intervention (Tuina + AZD8797) exerted a additive benefit, further elevating the nociceptive thresholds beyond those achieved by either monotherapy across days 7, 10, and 14 (*p* < 0.01, Figures 4B,C). Beyond behavioral recovery, transmission electron microscopy (TEM) corroborated these protective effects at the cellular level. Both Tuina and AZD8797 interventions effectively mitigated neuronal stress and damage, restoring pyknotic nuclei to a more typical morphology and normalizing the ultrastructural integrity of mitochondria and the endoplasmic reticulum. Notably, the combined treatment group (TA) exhibited the most profound ultrastructural preservation (Figure 4D).

### 3.5 Tuina preserves PNNs integrity and restores neuronal homeostasis by inhibiting excessive phagocytosis

Aberrant phagocytic activity is pivotal in mediating central neuroinflammation and tissue remodeling. To elucidate the regulatory effects of Tuina on phagocytic function and determine whether this translates to the structural preservation of PNNs, we quantified the protein levels of the phagocytosis and lysosome-associated markers TREM2 and Lamp1 via Western blotting, and evaluated the expression of WFA in the spinal dorsal horn via immunofluorescence.

**Figure 5:**
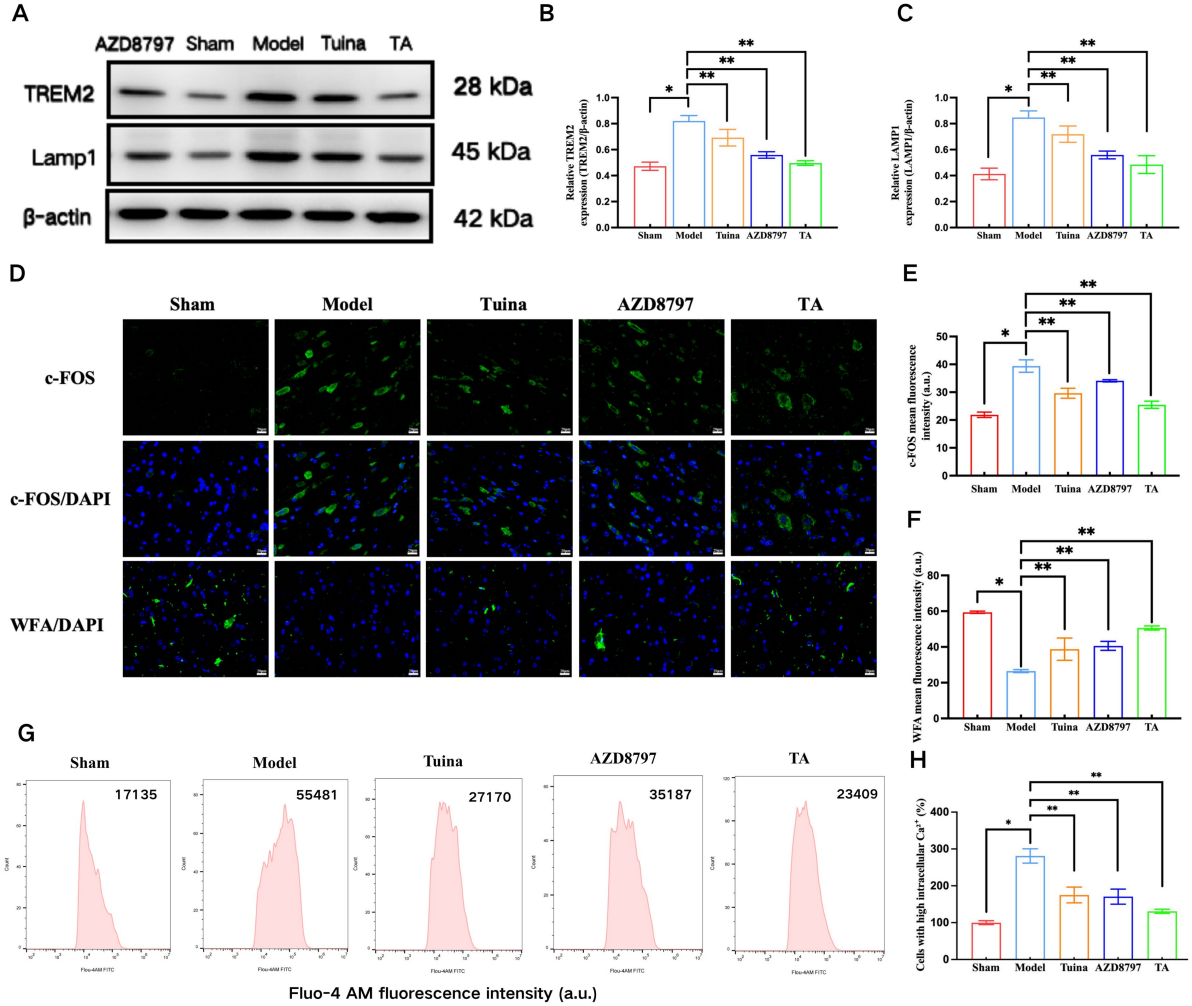
Tuina preserves PNNs integrity and restores neuronal homeostasis by inhibiting excessive phagocytosis. **(A)** Representative Western blot bands of phagocytosis-related proteins TREM2 and Lamp1 in the spinal dorsal horn across groups. *β*-Actin was used as an internal loading control. **(B,C)** Quantitative densitometric analysis of TREM2 **(B)** and Lamp1 **(C)** protein expression levels. **(D)** Representative parallel immunofluorescence images showing c-FOS (neuronal activation marker, green) and WFA (PNNs, green) with DAPI (nuclei, blue) counterstain in the spinal dorsal horn. Scale bar = 20 μm. **(E,F)** Quantitative analysis of c-FOS **(E)** and WFA **(F)** mean fluorescence intensities. **(G)** Representative flow cytometry plots showing intracellular Ca^2+^ levels (Fluo-4 a.m.). **(H)** Quantitative percentage of cells with high Ca^2+^ levels. Data are presented as mean ± SD (*n* = 8). *p < 0.05 vs. Sham group; **p < 0.05 vs. Model group. Statistical analysis was performed using one-way ANOVA followed by Tukey’s post-hoc test.

Western blot analysis suggest that Tuina significantly downregulated the expression of TREM2 and Lamp1 (*p* < 0.05, Figures 5A–C). Concurrently, immunofluorescence imaging and subsequent quantification revealed a significant reduction in WFA expression in the Model group compared to the Sham group (*p* < 0.05). Conversely, treatment with either Tuina or the inhibitor restored WFA levels relative to the Model group (*p* < 0.05, Figures 5D,F), with no statistical difference observed between the two monotherapies. Notably, the combined intervention exerted a further additive effect, elevating WFA expression beyond the levels achieved by Tuina alone (*p* < 0.05). The downregulation of TREM2 and Lamp1, coupled with the robust rebound in WFA intensity, indicates that Tuina potentially mitigates excessive phagocytic activity and associated with reduced degradation of PNNs.

The preservation of PNNs conferred subsequent neuroprotective benefits. Immunofluorescence analysis indicate that the aberrant overexpression of the neuronal activation marker c-FOS was significantly suppressed following Tuina intervention (*p* < 0.05, Figures 5D,E). Furthermore, flow cytometry confirmed that Tuina progressively mitigated intracellular Ca^2+^ accumulation in spinal cells (*p* < 0.05, Figures 5G,H).

### 3.6 Tuina exerts analgesia by inhibiting the upstream fractalkine/CX3CR1 and PI3K/AKT signaling pathways

**Figure 6:**
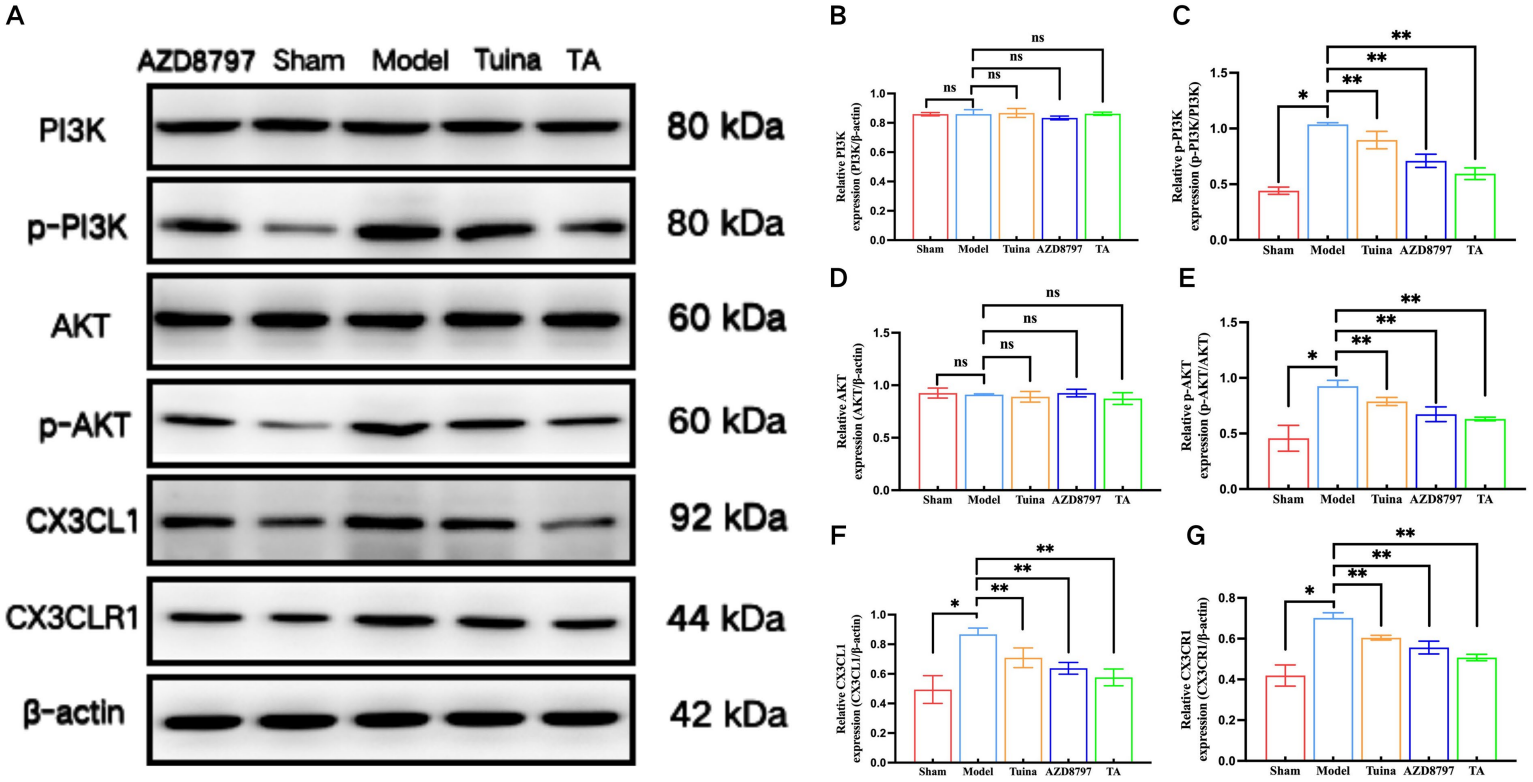
Tuina exerts analgesia by inhibiting the upstream Fractalkine/CX3CR1 and PI3K/AKT signaling pathways. **(A)** Representative Western blot bands for CX3CL1, CX3CR1, PI3K, p-PI3K, AKT, and p-AKT in the spinal dorsal horn across all five groups. **(B–G)** Quantitative densitometric analysis of CX3CL1, CX3CR1, and the ratios of p-PI3K/PI3K and p-AKT/AKT.β-Actin was used as the loading control. Data are presented as mean±SD (*n* = 8). *p < 0.05 vs. Sham group; **p < 0.05 vs. Model group; ^ns^*p* > 0.05. Statistical analysis was performed using one-way ANOVA followed by Tukey’s post-hoc test.

Finally, to elucidate the upstream molecular mechanisms orchestrating the aforementioned cellular and structural alterations, we interrogated the Fractalkine (CX3CL1)/CX3CR1 axis and the downstream PI3K/AKT pathway. Western blot analysis revealed that, compared to the Sham group, CCD modeling significantly upregulated CX3CL1 and CX3CR1 protein levels in the spinal dorsal horn (*p* < 0.05), accompanied by a sharp increase in the phosphorylation of PI3K and AKT (p-PI3K, p-AKT) (*p* < 0.05). Strikingly, Tuina intervention significantly suppressed the aberrant expression of CX3CL1 and CX3CR1, and robustly attenuated the levels of p-PI3K and p-AKT (*p* < 0.05). However, the total protein expression levels of PI3K and AKT showed no significant differences among the groups (*p* > 0.05, Figures 6A–G). This inhibitory effect closely mirrored the outcomes observed in the specific receptor antagonist AZD8797 group, compellingly suggesting that the ameliorative effects of Tuina on hyperalgesia in CCD rats are mediated through the targeted negative regulation of the CX3CL1/CX3CR1 signaling axis and its downstream PI3K/AKT phosphorylation cascade.

## 4 Discussion

Tuina, a traditional external therapy with a long history in China, is widely used to treat various conditions such as chronic pain, neurodegenerative diseases, cancer, immune disorders, cardiovascular diseases, sleep disorders, psychiatric disorders, dermatological conditions, pediatric cerebral palsy, and pediatric muscular torticollis (Field, 2016; Mak et al., 2024; Kong et al., 2025; Liu et al., 2025). The popularity of non-invasive therapies like Tuina for chronic pain is increasing both in China and globally (Qaseem et al., 2017; Ren et al., 2022). However, further research is needed to investigate the analgesic mechanisms of Tuina at the spinal level. In this study, rats in the Tuina group underwent CCD surgery followed by Tuina intervention at the ipsilateral Yinmen (BL37), Weizhong (BL40), and Chengshan (BL57) acupoints. The Model group underwent CCD surgery, while the Sham group received sham surgery. Rats in the AZD8797 and TA groups received intraperitoneal injections of AZD8797 (80 μg/kg) starting on day 4 post-surgery. The results showed that Tuina intervention significantly reduced mechanical allodynia and thermal hyperalgesia in the neuropathic pain (NP) model. Additionally, CCD surgery triggered microglial activation, PNNs degradation, and increased neuronal excitability, which were effectively reversed by Tuina treatment. In summary, our study indicates that neuropathic pain induced by CCD alters microglial phagocytic activity in the spinal dorsal horn, leading to PNNs degradation and abnormal transmission of nociceptive signals. Tuina therapy mitigates pain behavior by regulating microglial phagocytosis to prevent PNNs degradation.

A multinational randomized controlled trial (RCT) demonstrated that Tuina significantly reduces pain intensity and enhances quality of life, with sustained benefits observed at a 5-month follow-up, indicating its potential in managing chronic non-specific low back pain (Tan et al., 2026). Furthermore, a single-center, parallel-group RCT confirmed that a 6-week Tuina intervention was more effective than oral celecoxib in alleviating pain, improving negative affective states, and achieving long-term improvements in disability among patients with knee osteoarthritis (Xu et al., 2023). Additionally, a Chinese RCT reported that Tuina alleviates patellofemoral osteoarthritis (PFOA) by decreasing the degree of lateral patellar tilt, exhibiting efficacy comparable to that of intra-articular hyaluronic acid (IAHA) injection in pain relief (Gu et al., 2025). Despite this compelling clinical evidence, while previous studies have established the efficacy of Tuina in alleviating pain sensitization and inhibiting microglia-mediated neuroinflammation in NP, the underlying molecular mechanisms remain to be fully elucidated. Specifically, there is currently no conclusive evidence regarding whether Tuina exerts its analgesic effects by modulating the CX3CL1/CX3CR1 axis to alter microglial phagocytosis of PNNs, thereby preserving the homeostasis of the neuronal microenvironment in the spinal dorsal horn.

Modern research indicates that spinal dorsal horn neurons exhibit time- and region-dependent sensitization to repetitive noxious stimuli. Their sensitivity increases with disease duration, leading to intensified responses and difficulty in functional recovery (Ferrillo et al., 2022). CX3CL1 is an essential chemokine that regulates adhesion and chemotaxis by binding to its receptor, CX3CR1. In the central nervous system (CNS), CX3CR1 plays a critical role in the crosstalk between glial cells and neurons through direct or indirect mechanisms (Silva and Malcangio, 2021). The CX3CL1/CX3CR1 axis regulates microglial activation and function, neuronal survival, and synaptic function by controlling the release of inflammatory cytokines and synaptic plasticity during neurological diseases (Wang M. J. et al., 2024). Following Tuina intervention, the protein levels of CX3CL1 and CX3CR1 were significantly reduced, accompanied by a decrease in the expression of their downstream targets, p-AKT and p-PI3K. This indicates that Tuina exerts analgesic effects by inhibiting the CX3CL1/CX3CR1 pathway. These findings are consistent with numerous studies showing that modulating the CX3CL1/CX3CR1 axis in the spinal dorsal horn effectively inhibits pain sensitization in rats (Lyu et al., 2021; Subbarayan et al., 2022; Kubickova and Dubovy, 2024). Collectively, our results suggest that this chemokine axis may be a pivotal pathway underlying the central analgesic mechanism of Tuina.

In recent years, PNNs have emerged as a new focus in pain mechanism research (Dang et al., 2025; Mascio et al., 2025; Pang et al., 2025). As an extracellular matrix regulating neural plasticity, the integrity of PNNs is vital for maintaining neuronal excitability homeostasis.

Recent studies reveal that neuropathic pain involves region-specific pathological remodeling of PNNs in the spinal dorsal horn and cortical limbic system. Specifically, spinal PNNs degradation—driven by microglial phagocytosis or Cathepsin S release—leads to the loss of inhibitory tone and central sensitization, whereas aberrant PNNs accumulation in the thalamocortical circuit contributes to the consolidation of pain memory. Importantly, targeting the dynamic equilibrium of PNNs to restore neural network homeostasis has been proven to alleviate hyperalgesia and comorbid anxiety, presenting a promising extracellular matrix target for chronic pain therapy (Cermak et al., 2025; Mascio et al., 2025; Pang et al., 2025; Yang et al., 2025).

In this study, we evaluated the lysosome-associated proteins, phagocytic pathways, and the core structure of PNNs in the spinal dorsal horn. The results indicate that in the Tuina group, the expression levels of the phagocytosis-associated marker TREM2 and the lysosome-associated membrane protein Lamp1 were decreased, whereas WFA expression was increased. These findings indicate that Tuina inhibits the excessive phagocytic and lysosomal degradation capacity in the spinal dorsal horn. By attenuating this excessive phagocytosis, Tuina effectively reverses PNNs degradation, thereby providing a structural basis for restoring the homeostasis of inhibitory circuits in the spinal dorsal horn.

Crucially, given that CX3CR1 is predominantly expressed on microglia in the CNS, our findings strongly indicate that Tuina intervention effectively alleviates neuropathic pain by inhibiting the CX3CR1-mediated PI3K/AKT cascade. This pathway suppression likely curtails microglia-driven excessive phagocytosis (evidenced by decreased TREM2 and LAMP1), thereby preserving the structural integrity of PNNs to dampen central sensitization.

## 5 Limitations

This study has several limitations. First, the inherent differences between human clinical pain and the CCD rat model limit the direct generalizability of our findings. Second, this study primarily focused on the spinal level and did not explore the regulatory effects of Tuina on descending inhibitory pathways in the brainstem or the downstream regulatory networks of the PI3K/AKT pathway. Third, our evidence regarding microglial engulfment of PNN material remains indirect. Future studies employing specific microglial-depletion models and high-resolution direct imaging techniques are required to definitively confirm this direct physical interaction. To further strengthen the causal link between CX3CR1 signaling and PNN phagocytosis in the context of Tuina therapy, future studies should employ CX3CR1-Cre lineage tracing or microglial-specific knockouts, alongside a deeper exploration of PI3K/AKT downstream signaling networks. Furthermore, it is necessary to systematically investigate Tuina’s holistic regulatory effects on the ‘spinal cord-brainstem-cortex’ pain pathway. Lastly, because the single manipulation technique and fixed parameters used in our study may not fully reflect real-world efficacy, future clinical applications must ensure that manipulation types and stimulation intensities are tailored to individual patient conditions and tolerance.

## 6 Conclusion

**Figure 7:**
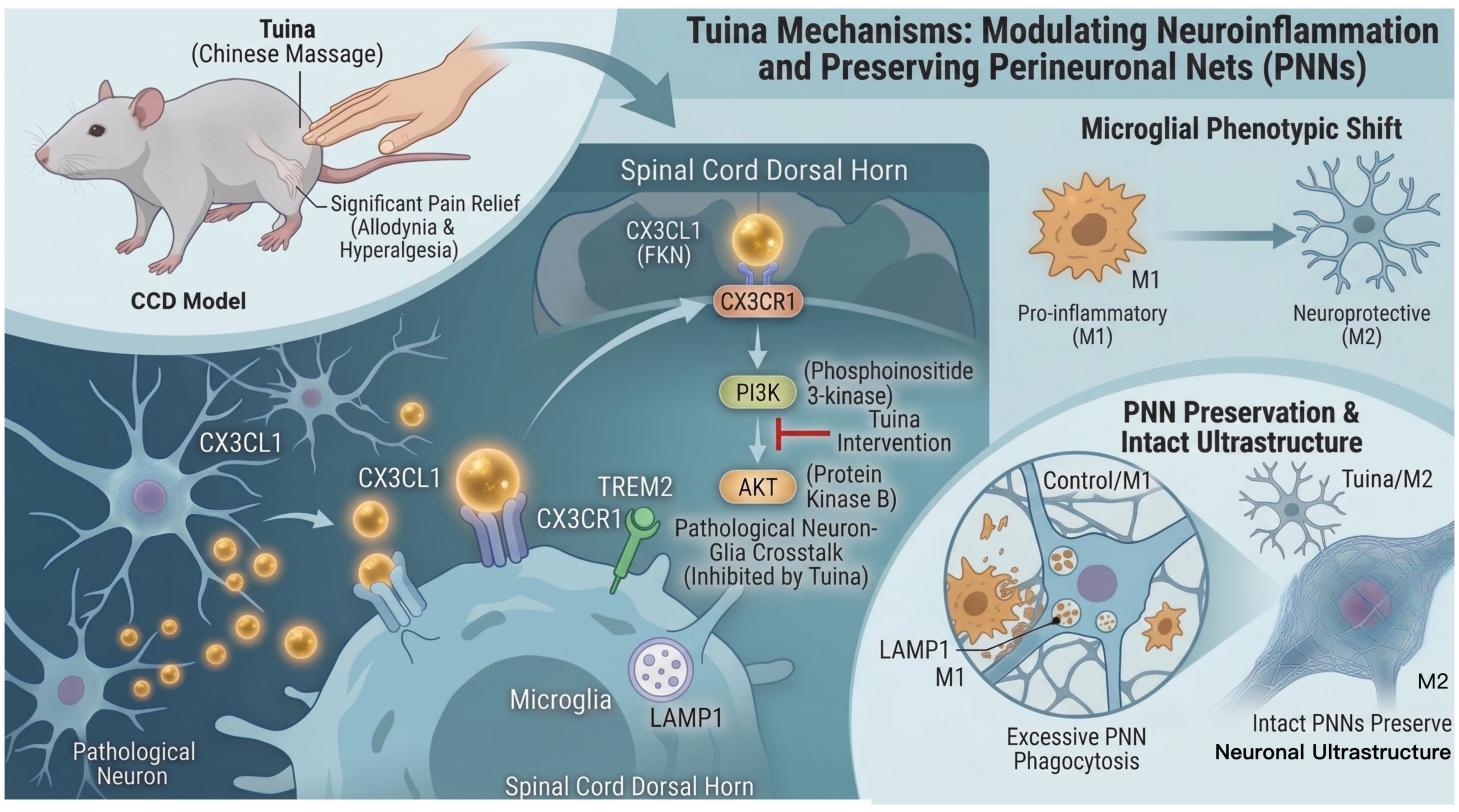
Schematic illustration of the proposed mechanism by which Tuina alleviates neuropathic pain.

In conclusion, the present study highlights the critical role of the CX3CL1/CX3CR1 signaling axis in mediating microglia-dependent degradation of perineuronal nets (PNNs) within the spinal dorsal horn following chronic compression of the dorsal root ganglion (CCD). Crucially, we suggest that Tuina intervention effectively alleviates neuropathic pain by inhibiting the CX3CR1-mediated PI3K/AKT cascade, thereby suppressing microglial excessive phagocytosis (evidenced by decreased TREM2 and LAMP1) and preserving the structural integrity of PNNs to dampen central sensitization. A schematic representation of this proposed mechanism is provided in Figure 7. These findings not only unravel a novel neuro-immune mechanism underlying the analgesic effects of Tuina but also propose a promising non-pharmacological strategy for the clinical management of chronic neuropathic pain.

## Data Availability

The raw data supporting the conclusions of this article will be made available by the authors, without undue reservation.
